# Bacterial Clearance Is Improved in Septic Mice by Platelet-Activating Factor-Acetylhydrolase (PAF-AH) Administration

**DOI:** 10.1371/journal.pone.0074567

**Published:** 2013-09-12

**Authors:** Mariana G. A. Teixeira-da-Cunha, Rachel N. Gomes, Nathassia Roehrs, Fernando A. Bozza, Stephen M. Prescott, Diana Stafforini, Guy A. Zimmerman, Patricia T. Bozza, Hugo C. Castro-Faria-Neto

**Affiliations:** 1 Laboratório De Imunofarmacologia, Instituto Oswaldo Cruz, Fiocruz, RJ, Brazil; 2 Laboratório de Investigação em Medicina Intensiva, IPEC, Fiocruz, RJ, Brazil; 3 Oklahoma Medical Research Foundation, Oklahoma City, United States of America; 4 Huntsman Cancer Institute, Salt Lake City, Utah, United States of America; 5 Program in Human Molecular Biology and Genetics, University of Utah, Salt Lake City, Utah, United States of America; University of California Los Angeles, United States of America

## Abstract

Current evidence indicates that dysregulation of the host inflammatory response to infectious agents is central to the mortality of patients with sepsis. Strategies to block inflammatory mediators such as PAF have been investigated as adjuvant therapies for sepsis. PAF-AH, the enzyme responsible for PAF degradation, showed positive results in pre-clinical studies and phase II clinical trials, but the results of a phase III study were disappointing. In this study, we investigated the potential protective mechanism of PAF-AH in sepsis using the murine model of cecal ligation and puncture (CLP). Treatment with rPAF-AH increased peritoneal fluid levels of the anti-inflammatory mediators MCP-1/CCL2 after CLP. The numbers of bacteria (CFU) in the peritoneal cavity were decreased in the rPAF-AH-treated group, indicating more efficient bacterial clearance after rPAF-AH treatment. Interestingly, we observed increased levels of nitric oxide (NO) after PAF-AH administration, and rPAF-AH treatment did not decrease CFU numbers either in iNOS-deficient mice or in CCR2-deficient mice. We concluded that administration of exogenous rPAF-AH reduced inflammatory injury, altered cytokine levels and favored bacterial clearance with a clear impact on mortality through modulation of MCP-1/CCL2 and NO levels in a clinically relevant sepsis model.

## Introduction

Sepsis is a major cause of death worldwide. Approximately 750,000 cases of sepsis occur annually in the USA, with a mortality rate of 28.6% [1], [2]. The rates of sepsis are increasing in hospitals worldwide. Using the US National Hospital Discharge Survey database, Martin et al. [3] reported an increase in the frequency of severe sepsis from 83 cases per 100,000 population in 1979 to 240 cases per 100,000 population in 2000.

Several studies have demonstrated the involvement of lipid mediators, such as platelet-activating factor (PAF), in a variety of human inflammatory syndromes and in animal models of diseases [4], [5]. High levels of PAF were found in plasma and tissues of animals subjected to animal models of sepsis as well as in patients with sepsis [5]–[8]. Circulating levels of endogenous PAF are controlled by PAF-acetylhydrolase (PAF-AH), which is a highly specific phospholipase that hydrolyzes the *sn*-2 acyl group of PAF and structurally related phospholipids, terminating their signals and thus regulating inflammatory responses [9], [10]. The anti-inflammatory properties of recombinant PAF-AH (rPAF-AH) in animal models were first shown after its cloning from human macrophages [11]. Clinical conditions of excessive inflammation leading to multiple organ failure are associated with suppressed levels of PAF-AH in the circulation and may explain the unchecked PAF activity and excessive inflammation in septic states [12]. In pre-clinical studies developed by our group, we observed that sepsis induced a significant decrease in plasma PAF-AH activity, and this effect correlated with the mortality of the animals subjected to endotoxemia and a clinically relevant model of sepsis, the cecal ligation and puncture (CLP) [13]. The effect of PAF-AH in clinical studies, however, is contradictory. Treatment with rPAF-AH resulted in positive effects on survival in a Phase II study with patients with sepsis or multiple traumas [14]. However, a Phase III trial failed to confirm this benefit [15]. The reasons for the difference in the Phase II and Phase III results remain to be determined and likely involve the multiple challenges inherent to sepsis trials.

In the present work, we further investigated the protective effect of rPAF-AH on animal models of sepsis, emphasizing the bacterial clearance and the mediators involved in this phenomenon. We have previously shown that PAF-AH treatment protects mice from polymicrobial sepsis, and this protective effect is accompanied by an increase in MCP-1 levels and is dependent on CCR2 signaling [13]. More recently, we have observed that MCP-1/CCL2 is important for optimal bacterial elimination, and this mechanism involves the release of nitric oxide, which is an important antimicrobial agent. The molecular pathway connecting an increase in MCP-1 levels with increased NO production, and therefore increased killing capacity, involves ERK activation [16]. Here, we show that administration of rPAF-AH alters cytokine levels and favors bacterial clearance through the modulation of MCP-1/CCL2 and NO levels.

## Materials and Methods

### Animals

Swiss and C57BL6 male mice weighing 20 to 25 g, age 6–8 weeks, from the Oswaldo Cruz Foundation breeding unit were used. The animals were maintained at a constant temperature (25°C) with free access to chow and water in a room with a 12-h light/dark cycle. In one set of experiments, MCP-1/CCL2 receptor-deficient mice (CCR2−/−) or inducible nitric oxide synthase-deficient mice (iNOS−/−), in a C57BL/6 background, were used. This study was approved by the committee of animal use (CEUA), Oswaldo Cruz Foundation, with the license number LW-36/10.

### Materials

Human rPAF-AH (Pafase@) was provided by ICOS Corporation (Bothell, WA, USA) as an endotoxin free solution. PAF and SR27417 were from Sigma-Aldrich (USA), and Lyso-PAF was from Cayman Chemical (Michigan-USA). Murine recombinant MCP-1 was from Peprotech (Rocky Hill, NJ). Griess reagent was from Sigma-Aldrich (USA). Thiopental (Thionembutal) was from Abott Labs. do Brasil, LTDA, and ketamine were from Cristália (Brazil).

### Cecal Ligation and Puncture (CLP)

Mice were anesthetized with a mixture of Thiopental (40 mg/kg) and Ketamine (80 mg/kg) diluted in sterile saline and administered intraperitoneally (0.2 ml). Laparotomy was performed, and the cecum was exposed and carefully ligated below the ileocecal junction with care to avoid bowel obstruction. The cecum was punctured once with an 18-gauge needle and then gently squeezed to empty its contents through the puncture. The cecum was returned to the peritoneal cavity, and the abdominal muscle and skin incisions were closed in layers using a 3-0 nylon suture line. Immediately after the surgery, 0.5 ml of sterile saline was administered subcutaneously to the animals for volume resuscitation. Sham-operated mice were subjected to identical procedures except that ligation and puncture of the cecum were omitted. Animals subjected to CLP developed early signs of sepsis, including lethargy, piloerection, and diarrhea. Survival of mice subjected to CLP or sham injury was determined daily for 7 days. For the experiments with rPAF-AH, the animals were treated with 1 mg/kg (i.p) of the enzyme fifteen minutes after the CLP procedure.

### Bacteria Inoculation

Mice were stimulated intraperitoneally with *S.* Typhimurium *(*5×10^5^ bact/cavity) or *E. coli* (10^6^ bact/cavity). For these experiments, the *E. coli* ATCC25922 strain was grown in LB medium (10 g of bactotriptona, 5 g of yeast extract and 10 g of NaCl, pH 7, sterilized by autoclaving at 120°C for 30 min.) from a single colony. For studies of *Salmonella* infection, *Salmonella enterica* serovar Typhimurium ISC 5302/2004, a generous gift from the Department of Microbiology of the Instituto Oswaldo Cruz, were cultured in Luria-Bertani broth (Guria Broth Miller; Sigma-Aldrich) for 16–18 h at 37°C to obtain stationary growth phase cultures. The bacteria were then centrifuged (200 rpm) for 10 min at 4°C, and the pellets were resuspended in PBS to an OD of 0.1 at 660 nm, corresponding to 10^8^ CFU/ml. The animals were treated with 1 mg/kg (i.p) of rPAF-AH fifteen minutes after bacterial stimulation. After 6 hours, the peritoneal cavity was washed, and the lavage fluid was collected for determination of CFU and NO levels.

### MCP-1/CCL2 Measurement

The levels of MCP-1/CCL2 were evaluated using an ELISA method. Mice were killed in a carbon dioxide chamber at designated time points, and the peritoneal cavity was opened and rinsed with 3 ml of PBS solution. The supernatant fractions were used for immunoassays. All measurements were performed in duplicate following the manufacturer’s instructions (R&D systems Duo set kit).

### Determination of CFU

Twelve microliters of peritoneal lavage fluid from each mouse were placed on ice and serially diluted with sterile saline. Twelve microliters of each dilution were plated on agar plates and incubated overnight at 37°C after which the number of colonies was counted.

### Nitric Oxide Measurements

Mice were euthanized in a CO_2_ chamber at designated time points, and the peritoneal cavity was opened and rinsed with 3 ml of PBS. The peritoneal fluid or supernatant of the cell culture was collected and centrifuged for determination of nitrite levels using Griess reagent according to the manufacturer’s instructions (Sigma-Aldrich).

### Isolation of Peritoneal Macrophages

Peritoneal macrophages were obtained after washing the peritoneal cavity with 3 ml of PBS. One million cells were distributed on culture plates and placed into a 37°CO_2_ incubator for 2 h. All nonadherent cells were subsequently removed, and the adherent cells were treated with rMCP-1/CCL2 (100 ng/ml) or rPAF-AH (0.025 mg/well) and stimulated with *E. coli* (10^5^ bact./well). After 1 hour of *E. coli* stimulation, the supernatant was obtained and used for nitrite determination by the Griess assay as described above.

### Statistical Analysis

Data were analyzed statistically using the unpaired t-test with the Graph Pad Prism software. Data were considered statistically significant if *P* values were less than 0.05.

## Results

### Treatment with PAF-AH Increases Bacterial Clearance in the Peritoneal Cavity of Animals Subjected to CLP or Inoculated with Salmonella *Typhimurium*

**Figure 1:**
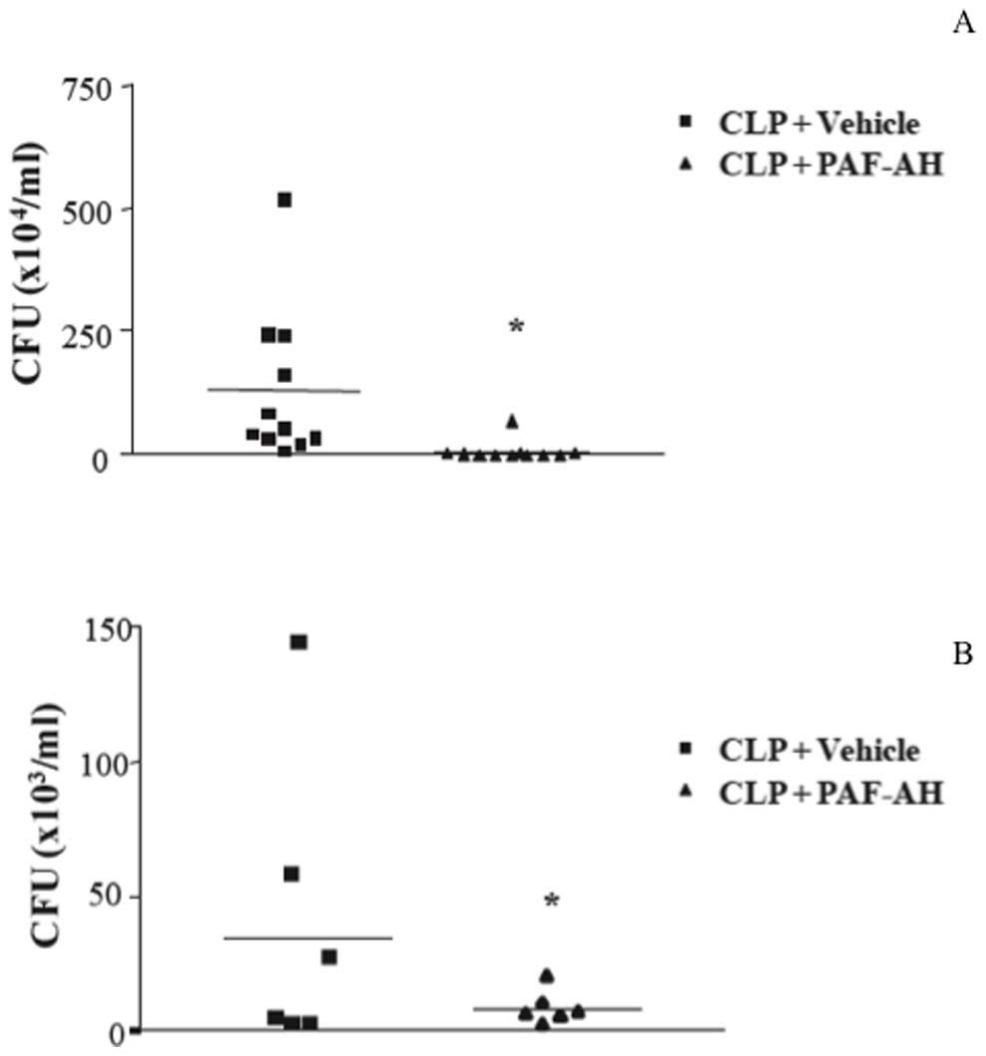
Treatment with rPAF-AH decreases CFU numbers in the peritoneal fluid after CLP model. (A) Mice subjected to CLP were treated with rPAF-AH (1 mg/kg-i.p. 15 minutes after). At 6 h after CLP, the mice were euthanized, and the peritoneal fluid was collected and plated on agar plates. (B) At 24 h after CLP, mice were euthanized, and the peritoneal fluid was collected and plated on agar plates. After infection, mice were euthanized 6 (CLP+Vehicle n = 11 animals; CLP+PAF-AH n = 10) and 24 (CLP+Vehicle n = 6 animals; CLP+PAF-AH n = 6) hours after infection, and the peritoneal fluid was collected. Twelve microliters of the peritoneal fluid from each mouse were serially diluted and plated on agar plates. The data represent three different experiments, and the horizontal line represents the median of CFU count. (*) indicate significant difference (P = 0.05) when compared to vehicle treatment.

**Figure 2:**
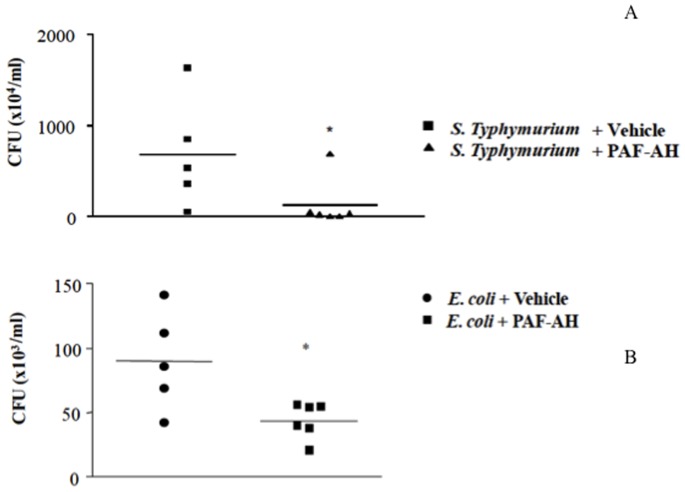
Treatment with rPAF-AH decreases CFU numbers in the peritoneal fluid after *S.* Typhimurium *or E. coli* administration. (A) Mice injected with *S.* Typhimurium *(5×10^5^)* were treated with rPAF-AH (1 mg/kg-i.p. 15 minutes after - *S.* Typhimurium+Vehicle n = 5 animals; *S.* Typhimurium+PAF-AH n = 6). (B) Mice injected with *E. coli (10^6^)* were treated with rPAF-AH (1 mg/kg-i.p. 15 minutes after- *E. coli*+Vehicle n = 5 animals; *E. coli* +PAF-AH n = 4). Six hours after infection, the mice were euthanized, and the peritoneal fluid was collected. Twelve microliters of the peritoneal fluid from each mouse were serially diluted and plated on agar plates. The data represent three different experiments, and the horizontal line represents the median of CFU count. (*) indicate significant difference (P = 0.05) when compared to vehicle treatment.

Previous studies demonstrated that administration of rPAF-AH (1 mg/kg) 15 min or 6 h after CLP increased the survival rate and reduced the inflammatory response [13]. To investigate the mechanisms involved in this protective effect, we examined the numbers of bacterial colony forming units (CFU) in the peritoneal cavity of the animals after treatment in different models of sepsis. As shown in Figures 1A and B, CFU numbers were significantly diminished in PAF-AH-treated groups 6 and 24 hours after the CLP procedure. Because CLP is a polymicrobial model of sepsis, we investigated if rPAF-AH would increase bacterial clearance in a model of Gram-negative sepsis induced by intraperitoneal injection of *S.* Typhimurium or *E. coli*. As shown in Figures 2A and B, rPAF-AH treatment decreased CFU numbers in both models of bacterial infection.

To investigate a direct anti-bacterial effect of rPAF-AH, we incubated *S.* Typhimurium or *E. coli* with rPAF-AH in the absence of host cells. We observed that incubation with rPAF-AH did not affect bacterial growth of *S.* Typhimurium (721.3±20.9-vehicle versus 731.0±29.7-PAF-AH group) or *E. coli* (344.8±144.0-vehicle versus 259.0±131.7-PAF-AH group). Similar results were observed when we investigated the direct effect of rPAF-AH on Gram-positive bacteria *S. aureus* (344.8±144.0-vehicle versus 259.0±131.7-PAF-AH group). The hydrolysis of PAF by PAF-AH releases the biologically inactive PAF metabolite, lyso-PAF. Therefore, we investigated if PAF or lyso-PAF had any direct effect on bacterial growth. We observed no difference in the CFU number after incubation of *S.* Typhimurium with PAF or lyso-PAF (539.1±62.5-vehicle versus 505.2±19.7-PAF versus 491.0±29.1-Lyso-PAF group).

### Treatment with rPAF-AH Increases the Production of MCP-1/CCL2 and Nitric Oxide in Septic Mice

**Figure 3:**
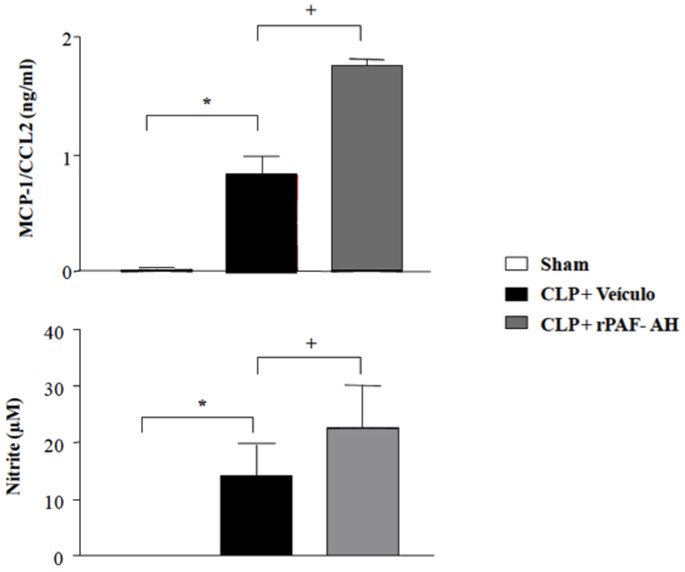
Treatment with rPAF-AH increases MCP-1/CCL2 and NO levels in the peritoneal cavity after CLP. Swiss mice were injected intraperitoneally with rPAF-AH (1 mg/kg) or vehicle 15 min after the CLP procedure (n = 6 in all groups). After 6 h, the peritoneal fluid was obtained and used for MCP-1/CCL2 (A) or NO (B) determination by ELISA and Griess assays, respectively. The values are the mean±SEM from 6 animals. (*) Statistically significant differences when compared to Sham group. (+) Statistically significant differences when compared to CLP+vehicle group.

**Figure 4:**
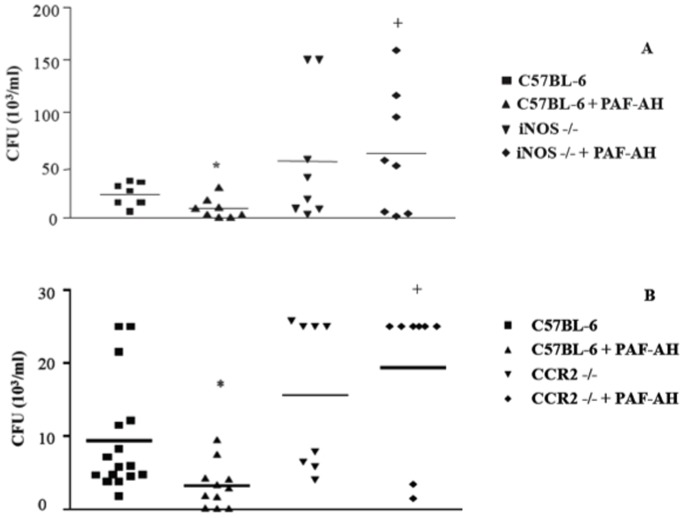
CFU numbers are not affected by rPAF-AH treatment in MCP-1/CCR2-deficient mice (CCR2−/−) or iNOS-deficient mice (iNOS−/−) subjected to CLP. Mice were treated with rPAF-AH (1 mg/kg-i.p. - minimum of 7 animals in each group) 15 min after CLP. At 6 h, the mice were euthanized, and the peritoneal fluid was plated on agar plates. The data represent three different experiments, and the horizontal line represents the median of CFU count. (*) Statistically significant differences when compared with C57BL-6 group. (+) Statistically significant differences when compared with C57BL-6+PAF-AH group.

To analyze the mediators involved in bacterial clearance, we measured the MCP-1/CCL2 and nitric oxide (NO) levels in the peritoneal cavity of the animals 6 hours after CLP. Several studies have demonstrated the involvement of NO as a bactericide substance and its central role in phagocytosis [17]–[21]. As shown in Figures 3A and B, treatment of septic mice with rPAF-AH significantly increased MCP-1/CCL2 and NO levels in the peritoneal fluid of treated mice compared to the control. We next used mice deficient in inducible nitric oxide synthase (iNOS−/−) to assess if NO was involved in rPAF-AH-induced bacterial clearance. We found that administration of rPAF-AH to iNOS−/− mice failed to reduce the CFU numbers when compared to the vehicle-treated group. The CFU numbers were significantly higher in rPAF-AH-treated iNOS−/− mice than in wild-type mice, further reinforcing the role of NO in controlling bacterial clearance in the model (Figure 4A).

### MCP-1/CCL2 Levels are Relevant to NO Production and Increased Bacterial Clearance after Treatment with rPAF-AH

We have previously demonstrated that rPAF-AH treatment alters the cytokine/chemokine profile in sepsis [13]. We hypothesized that these changes in the cytokine/chemokine profile, specifically increased MCP-1 levels, would favor bacterial clearance by the host. Here we confirmed that treatment with rPAF-AH increased MCP-1 levels in the peritoneal fluid 6 hours after CLP. Nakano et al. have demonstrated that treatment with MCP-1 protected the animals from mortality induced by *P. aeruginosa* by increasing the bacterial clearance [22]. To investigate if increased MCP-1/CCL2 levels would be important for more efficient clearance of bacteria after rPAF-AH treatment, we performed CLP in MCP-1/CCL2 receptor-deficient mice (CCR2−/−). As observed in iNOS−/− mice, we found that rPAF-AH treatment did not affect CFU numbers in CCR2−/− mice (Figure 4B). Interestingly, treatment with rPAF-AH did not increase survival in CCR2−/− mice (data not shown) [13]. To investigate a direct anti-bacterial effect of MCP-1/CCL2, we incubated *E. coli* with MCP-1/CCL2 in the absence of host cells. We observed that incubation with MCP-1/CCL2 did not affect *E. coli* bacterial growth (372±44,2- *E.coli*+vehicle versus 400±42,5 *E.coli*+MCP-1).

**Figure 5:**
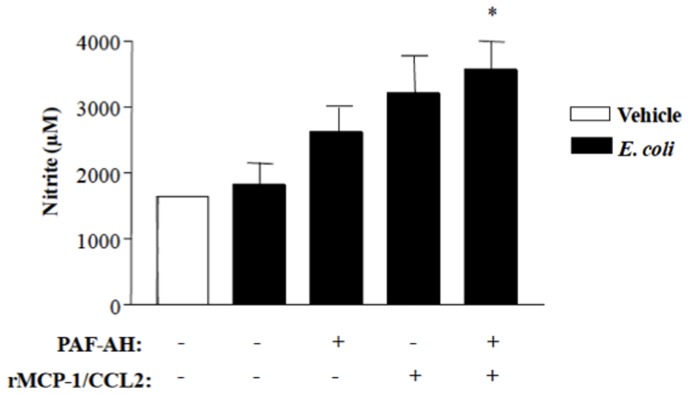
rPAF-AH and MCP-1/CCL2 increase NO production by peritoneal macrophages stimulated with *E. coli*. Peritoneal macrophages (10^6^ cells/well) were incubated with rMCP-1 (100 ng/ml), PAF-AH (0,025 mg/well) or rMCP-1+PAF-AH and stimulated with *E. coli* (10^5^ bact./well). After 1 hour of *E. coli* stimulation, the supernatant was obtained and used for nitrite determination by the Griess assay. The values are the mean±SEM from 4 wells. The data represent two different experiments. (*) Statistically significant differences when compared with *E. coli* group (P = 0.030).

To further demonstrate the interaction between MCP-1/CCL2 and NO, we showed that MCP-1/CCL2 and PAF-AH had an additional effect on NO production when peritoneal macrophages were stimulated with *E. coli* (Figure 5). Altogether, these results indicate that the increase in MCP-1/CCL2 and NO levels induced by the treatment of septic mice with rPAF-AH leads to a more efficient bacterial clearance at the site of infection and therefore impacts the beneficial effects of this enzyme in septic conditions.

## Discussion

The killing of infectious agents is an essential component of the first-line host defense and a central mechanism in the host immune innate response against pathogens [23], [24]. The importance of this phenomenon in the development of sepsis has been clearly demonstrated [25], [26]. In the present study, we characterized a previously unknown effect of PAF-acetylhydrolase in bacterial clearance in a clinically relevant model of sepsis. Previous studies from our laboratory have shown that rPAF-AH treatment protects animals from sepsis, modifies cytokine levels and decreases leukocyte accumulation and activation. Although the precise molecular mechanism for this protective effect is not completely understood, our previous study suggests the involvement of the chemokine MCP-1/CCL2 [13].

PAF signaling influences bacterial clearance in different models. Moreno et al. (2006) demonstrated an improvement of bacterial clearance and a reduction of the systemic spread of bacteria after treatment with the PAF receptor antagonist PCA-4248 in mice subjected to CLP [27]. In addition, treatment with another PAF receptor antagonist, WEB2170, induced protection with more effective bacterial clearance in the lungs of mice infected with *Klebisiella pneumoniae* [28]. Studies with PAF receptor-deficient mice (PAFR−/−) demonstrated that deficient PAFR signaling is associated with protection against lethal pneumonia, attenuated bacterial growth in the lungs and diminished dissemination of the infection after infection with *S. pneumoniae* [29]. Our observations that rPAF-AH treatment decreases CFU count numbers in animals subjected to CLP or inoculated with *Salmonella* Typhimurium further indicate that decreasing PAF signaling modulates innate responses to improve bacterial killing in septic conditions. A direct effect of rPAF-AH in bacterial growth and/or viability, although unlikely, could explain our observations in the CLP or *S.* Typhimurium model. Nevertheless, the results from incubating *S.* Typhimurium, *E. coli* or *S. aureus* (data not shown) with rPAF-AH *in vitro* completely eliminated this possibility. A polymicrobial infection and a Gram-negative infection (*S.* Typhymurium) were similarly affected by treatment with rPAF-AH, indicating a specific effect restricted to one or few bacterial species and that the increase in bacterial clearance induced by rPAF-AH is a more general process involving basic innate immune response pathways.

PAF is an early mediator of inflammatory syndromes, such as sepsis, that can influence cytokine response and cell behavior [30], [31]. In a previous study, we measured the cytokine profile in the peritoneal fluid of mice subjected to CLP after treatment with rPAF-AH. We observed that rPAF-AH treatment decreased the levels of KC, IL-6, TNF and MIF while it increased the levels of anti-inflammatory cytokines, such as IL-10 and MCP-1/CCL2 [13]. MCP-1/CCL2 has a protective effect in murine endotoxemia [32], and administration of blocking monoclonal antibodies [33] or genetic deficiency of MCP-1/CCL2 is associated with increased mortality rates in mice with sepsis [34]. Increased levels of MCP-1/CCL2 after rPAF-AH treatment in septic mice are associated with decreased mortality and, in fact, rPAF-AH failed to decrease mortality in animals that were genetically deficient in the MCP-1/CCL2 receptor (CCR2−/−) [13]. The precise molecular mechanism for this protective effect is not completely understood; however, in the present investigation, we postulated that changes in the cytokine/chemokine profile, especially MCP-1 levels, modulated innate responses to favor bacterial clearance and hence control infection. We found evidence to support this hypothesis because CFU numbers were decreased in the peritoneal fluid of mice subjected to CLP or challenged with *S.* Typhimurium and treated with rPAF-AH, while this effect was abrogated in CCR2−/− mice.

Nitric oxide (NO) has numerous physiological and pathophysiological actions, including both pro-inflammatory and anti-inflammatory properties depending on the phase of the inflammatory reaction and the cellular population responsible for its production [35]. In addition, NO plays a crucial role in killing pathogens including Gram-positive and Gram-negative bacteria [20], [21], [36]–[38]. In fact, iNOS−/− mice are more susceptible to CLP due to their inability to control the growth of bacteria at the infection site [39]. We measured the levels of NO in the peritoneal fluid of mice submitted to CLP and treated with rPAF-AH and observed increased levels when compared to controls, suggesting that the increased bacterial clearance after rPAF-AH treatment resulted from increased NO production under this condition. Confirming that NO is involved in microbicidal activity, the treatment with rPAF-AH was not effective in reducing bacterial CFU in the peritoneal fluid after CLP in iNOS−/− mice.

Several studies indicate the involvement of MCP-1/CCL2 in bacterial clearance. For instance, pretreatment of mice with anti-MCP-1/CCL2 increased the lethality rate and was associated with impaired bacterial clearance in a model of peritoneal sepsis [33]. In addition, treatment with rMCP-1/CCL2 increased bacterial clearance and protected mice after infection with *Pseudomonas aeruginosa* or *Salmonella* Typhimurium [22]. MCP-1/CCL2-deficient mice infected with *Salmonella* Typhimurium showed increased mortality and bacterial dissemination, characterized by pathogens in the liver and spleen [40]. Our results concur with these studies because rPAF-AH treatment increased bacterial clearance in parallel with an increase in MCP-1 levels but failed in CCR2−/− mice. This finding suggests an important role for MCP-1/CCL2 and its signaling through CCR2 in the control of bacterial burden during infection.

Cytokines influence the production of NO by leukocytes through modulating the expression of iNOS [41]. NO and MCP-1 co-regulation in a number of endotoxin-activated cells, including macrophages, has been investigated. Experiments demonstrated that LPS-induced production of the CC chemokines MCP-1 and MIP-1 was NO dependent [42]. In another study, murine macrophages activated with tGPI-mucin in the presence of IFN-γ produced large quantities of NO and CCL2. On the other hand, MCP-1 exerts a number of effects via the increase or decrease in the production of NO by activated cells [43], [44]. For instance, MCP-1/CCL2 contributes to the production of NO by macrophages infected with *T. cruzi* and hence to the microbiostatic activity of activated macrophages [45]. Our results show that synergism for NO production was also observed when macrophages stimulated with *E. coli* were treated with rPAF-AH and MCP-1, which correlated with better bacterial killing by macrophages.

Our results identify, for the first time, a fundamental role for rPAF-AH treatment in bacterial clearance, which may contribute to the beneficial effect of this enzyme observed in sepsis models [13] and in defined conditions in the clinical setting (21). In addition, we identified a synergistic mechanism involving MCP-1 and NO production that is under control of PAF signaling, and therefore affected by rPAF-AH treatment, and bolsters the ability of macrophages to kill invading bacteria. These findings may lead to additional discussion about the therapeutic potential of rPAF-AH in patients with sepsis.
